# Bariatric Surgery in Adolescents: To Do or Not to Do?

**DOI:** 10.3390/children8060453

**Published:** 2021-05-27

**Authors:** Valeria Calcaterra, Hellas Cena, Gloria Pelizzo, Debora Porri, Corrado Regalbuto, Federica Vinci, Francesca Destro, Elettra Vestri, Elvira Verduci, Alessandra Bosetti, Gianvincenzo Zuccotti, Fatima Cody Stanford

**Affiliations:** 1 Pediatric and Adolescent Unit, Department of Internal Medicine, University of Pavia, 27100 Pavia, Italy; valeria.calcaterra@unipv.it; 2 Pediatric Department, “V. Buzzi” Children’s Hospital, 20154 Milan, Italy; elvira.verduci@unimi.it (E.V.); alessandra.bosetti@asst-fbf-sacco.it (A.B.); GianVincenzo.Zuccotti@unimi.it (G.Z.); 3 Clinical Nutrition and Dietetics Service, Unit of Internal Medicine and Endocrinology, ICS Maugeri IRCCS, 27100 Pavia, Italy; hellas.cena@unipv.it (H.C.); Debora.porri01@universitadipavia.it (D.P.); 4 Laboratory of Dietetics and Clinical Nutrition, Department of Public Health, Experimental and Forensic Medicine, University of Pavia, 27100 Pavia, Italy; 5 Pediatric Surgery Department, “V. Buzzi” Children’s Hospital, 20154 Milan, Italy; francesca.destro@asst-fbf-sacco.it (F.D.); elettra.vestri@asst-fbf-sacco.it (E.V.); 6 Pediatric Unit, Fond. IRCCS Policlinico S. Matteo and University of Pavia, 27100 Pavia, Italy; corrado.regalbuto01@universitadipavia.it (C.R.); fede90vinci@gmail.com (F.V.); 7 Department of Health Sciences, University of Milan, 20146 Milan, Italy; 8 “L. Sacco” Department of Biomedical and Clinical Science, University of Milan, 20146 Milan, Italy; 9 Massachusetts General Hospital and Harvard Medical School, Boston, MA 02114, USA; fstanford@mgh.harvard.edu

**Keywords:** pediatric obesity, bariatric surgery, adolescents, nutritional status, weight loss, laparoscopic sleeve gastrectomy, multi-disciplinarity, complications

## Abstract

Pediatric obesity is a multifaceted disease that can impact physical and mental health. It is a complex condition that interweaves biological, developmental, environmental, behavioral, and genetic factors. In most cases lifestyle and behavioral modification as well as medical treatment led to poor short-term weight reduction and long-term failure. Thus, bariatric surgery should be considered in adolescents with moderate to severe obesity who have previously participated in lifestyle interventions with unsuccessful outcomes. In particular, laparoscopic sleeve gastrectomy is considered the most commonly performed bariatric surgery worldwide. The procedure is safe and feasible. The efficacy of this weight loss surgical procedure has been demonstrated in pediatric age. Nevertheless, there are barriers at the patient, provider, and health system levels, to be removed. First and foremost, more efforts must be made to prevent decline in nutritional status that is frequent after bariatric surgery, and to avoid inadequate weight loss and weight regain, ensuring successful long-term treatment and allowing healthy growth. In this narrative review, we considered the rationale behind surgical treatment options, outcomes, and clinical indications in adolescents with severe obesity, focusing on LSG, nutritional management, and resolution of metabolic comorbidities.

## 1. Introduction

Recent data on obesity prevalence in youth present significant concerns. According to the World Health Organization (WHO), over 340 million children and adolescents aged between 5−19 years experienced overweight and obesity in 2016; moreover, data from the National Health and Nutrition Examination Survey (NHANES) [1] in the USA demonstrate high values: 16.1% of young people aged 2 to 19 are classified as overweight, 19.3% with obesity and 6.1% with class III obesity (severe obesity) [2]. Pediatric obesity is a multifaceted disease that can impact physical and mental health [2,3], a complex condition that interweaves biological, developmental, environmental, behavioral, and genetic factors, as with adults. Pediatric obesity is associated with a greater risk for premature mortality and earlier onset of chronic disorders such as Type 2 Diabetes [4], dyslipidemia [5], nonalcoholic fatty liver disease (NAFLD) [6], obstructive sleep apnea (OSA) [7], and polycystic ovary syndrome (PCOS) [8], and adolescents with obesity are at increased risk of psychological disturbances.

In most cases diet, lifestyle modifications, and currently available pharmaceutical agents are relatively ineffective in treating severe obesity in the long term [9]. Thus, bariatric surgery has become a therapeutic strategy in adolescents [10] with an increased number of surgical procedures in Europe [11], the United States [12] and beyond [13].

In particular, laparoscopic sleeve gastrectomy (LSG) has been considered an accepted stand-alone bariatric surgery procedure. As in adults, this surgical treatment is safe and effective for patients under 18 years, leading to significant weight loss, remission of comorbidities, and improvement of quality of life (QoL) [13,14]. Multidisciplinary interventions are mandatory for the care of adolescents with severe obesity. Special attention should be given to optimize nutritional diagnosis and intervention prior to and after surgery.

In this narrative review, we consider the rationale behind surgical treatment options, outcomes, and clinical indications in adolescents with severe obesity, with particular focus on LSG, nutritional management, and resolution of metabolic comorbidities. 

## 2. Methods

Each author identified and critically reviewed the most relevant published studies (original papers and reviews) in the scientific literature. Papers published up to November 2020 in each author’s field of expertise were searched with the following keywords: obesity, adolescents, obesity complications, metabolic risk, bariatric surgery, sleeve gastrectomy, resolution of comorbidities, clinical indications for bariatric surgery. The following electronic databases were searched: PubMed, Scopus, EMBASE and Web of Science. The contributions were collected, and the resulting draft was discussed among authors to provide a theoretical point of view. The final version was then recirculated and approved by all the co-authors.

## 3. Obesity, Cardiometabolic Complications and Medical Treatment

Childhood obesity represents a troublesome public health problem which affects the majority of developed countries [1]. There are currently three major classifications used to assess overweight or obesity in children/adolescents. The cut-off points are based on growth curves according to the World Health Organization (WHO), the International Obesity Task Force (IOTF), and the US Centers for Disease Control (CDC). Concerning WHO classification, children aged between 5–19 years are classified as overweight or with obesity when body mass index (BMI)-for-age and sex is at or above the 85th percentile and below the 97th percentile, or above the 97th percentile, respectively [15]. According to CDC overweight is defined as a BMI at or above the 85th percentile and below the 95th percentile for children and teens of the same age and gender; obesity is defined as a BMI at or above the 95th percentile [16]. The IOTF system uses smooth gender-specific BMI curves, constructed to match the values of ≥25 kg/m^2^ (Overweight) and ≥30 kg/m^2^ (Obesity) at 18 years, thus providing age and gender BMI cut-offs for overweight and obesity, based on large data sets from six countries or regions covering different races/ethnicities [17].

It is well known that obesity-related complications and diseases are numerous, including metabolic and cardiovascular complications (Table 1).

Metabolic complications develop early in children and adolescents with obesity and worsen as the obesity degree increases. In addition, the prevalence of metabolic syndrome (MetS) in children and adolescents has increased with increasing prevalence of obesity [19]. MetS refers to a clustering of co-incident and inter-related risk factors that place an individual at high risk of developing cardiovascular disease and type 2 diabetes with increased mortality risk.

In the scientific literature, there are currently no standardized diagnostic criteria for MetS in pediatrics. As reported in Table 2, different classifications have been proposed; thus, a wide range of MetS prevalence rates is reported.

In a recent review by Reisinger et al. [22], the prevalence of MetS in pediatric age ranged from 0.3% to 26.4%. The lowest prevalence (0.3%) was found, according to the IDF definition [23], in the Colombian pediatric population, whereas the highest prevalence (26.4%) was observed among Iranian children [24] and adolescents according to the criteria of de Ferranti et al. The median prevalence value of the entire dataset was 3.8%. These data have to be seriously considered in order to assess the potential future health risk, taking into account the young age [25] of the examined subjects. Children with MetS have an increased risk of continued MetS in adulthood with a high likelihood of type 2 diabetes mellitus and cardiovascular disease [26]. For this reason, it is necessary to intervene decisively and effectively obesity in adolescents to prevent future related health complications and impaired quality of life.

It is clear that the first step in the treatment of obesity and metabolic syndrome in children is lifestyle medicine by means of dietary counseling, physical activity, and behavioral changes. The Endocrine Society Clinical Practice Guidelines recommend a minimum of 20 min of moderate-to-vigorous physical activity daily, independent of the grade of adiposity, in order to obtain weight loss and improve insulin sensitivity by counteracting the insulin resistance secondary to obesity [27,28,29].

In addition, a balanced and high-fiber diet is strongly recommended and appears to correlate with increased peripheral insulin sensitivity [30,31] lower risk of developing MetS in children and adolescents, lower systolic blood pressure and fasting blood glucose [32], as well as a healthier composition and diversity of gut microbiome, which may affect nutrient metabolism and energy balance [33]. In contrast, many studies have shown that high fat intake impairs insulin-sensitivity [34,35] regardless of adolescents’ adiposity [36]. Moreover, if high intake of saturated fats is also accompanied by excessive intake of refined grains, simple sugars, salt, and inadequate intake of fiber, as in the Western diet [37,38] this promotes inflammation [38] and changes of the gut microbiome profile, from healthy to a pattern more common in obesity [39,40]. The Western diet also influences the development of hypertension; the American Academy of Paediatric (AAP) recommends adoption of the Dietary Approaches to Stop Hypertension (DASH), which includes a diet rich in fruits, vegetables, low-fat dairy products, whole grains, fish, poultry, nuts, lean red meat and low in sugar, sweets, and sodium, for children and adolescents with hypertension [41]. If necessary, in addition to lifestyle and dietary modifications, prescription of approved medications for weight loss can be recommended. 

At the moment, there are no singular effective medical strategies available for long-lasting weight reduction in adolescents with severe obesity. Weight loss medications, while effective, have low popularity, are cost prohibitive as they are not covered by National Health Care, and there are safety concerns due to historical issues associated with weight loss drugs [41]. Moreover 3–44% of patients on weight-loss medication may experience side effects [42,43]. However, recent data on the use of weight loss medications shows promise in the pediatrics population [44,45].

Approved pharmacological treatments for obesity in pediatric age are limited. Orlistat, which acts as an inhibitor of intestinal lipase for adolescents aged ≥12; phentermine, a sympathomimetic amine, approved in teenagers aged ≥16 years and liraglutide, a glucagon-like peptide-1 receptor. (GLP-1) agonist, in pediatric (7–11 years) have been approved by the Food and Drug Administration (FDA). Liraglutide was also approved this year by the European Medicines Agency (EMA) in 12–17 old children [46].

For the treatment of insulin resistance, pharmacological intervention in pediatric age consists of off-label drugs use, since no drug has been specifically approved for this population. Metformin, a biguanide, represents the first-choice medication. It is administered orally and acts to reduce glucose levels, inhibiting the process of hepatic gluconeogenesis and promoting intestinal absorption of glucose [47,48,49]. Although metformin does not often result in significant body weight loss, it appears to prevent or delay alteration of glucose homeostasis in children at high risk of developing type 2 diabetes mellitus [50]. There are studies showing that metformin improves insulin sensitivity in adolescents with type 2 diabetes and polycystic ovary syndrome (PCOS) [51].

In addition to metabolic irregularities, there are cardiovascular irregularities such as dyslipidemia which warrant early diagnosis and management [52]. The treatment of dyslipidemia in childhood starts with lifestyle modification: low saturated fat and simple sugars dietary intake, adequate physical exercise and, if necessary, weight reduction. The AAP recommends prescription of medications (along with lifestyle modifications) in patients 8 years or older with LDL cholesterol (LDL-C) ≥190 mg/dL, or ≥160 mg/dL if there is a positive family history of premature cardiovascular disease and/or presence of other risk factors, also when LDL-C is ≥130 mg/dL if there is diabetes mellitus. For children younger than 8 years of age, the use of medication is only recommended when LDL-C values are ≥500 mg/dL [53]. According to the National Heart Lung and Blood Institute (NHLBI) children younger than 10 years of age should not be treated pharmacologically unless they have severe primary hyperlipidemia or high-risk condition associated with severe medical morbidity (homozygous hypercholesterolemia, LDL cholesterol level ≥400 mg/dL, primary hypertriglyceridemia with a triglyceride level ≥500 mg/dL, and cardiovascular disease evident in the first 2 years of life after cardiac transplantation). It is also necessary to initiate drug treatment in children older than 10 years, if LDL cholesterol levels consistently exceed 190 mg/dL, after a 6-months lifestyle intervention attempt [53,54]. Statins, HMG-CoA reductase inhibitors, are recommended as first-line approach in pediatric patients [54]. 

With regards to hypertension, the AAP Clinical Practice guidelines for screening and management of high blood pressure in children and adolescents, published in 2019, recommend initiating drug therapy with a single medication for children remaining hypertensive despite lifestyle modifications, or who have symptomatic hypertension, stage 2 hypertension without a clearly modifiable factor (e.g., obesity), or any stage of hypertension associated with type 1 diabetes mellitus or chronic kidney disease [53,55]. Recommended pharmacologic treatment includes the use of angiotensin-converting enzyme (ACE) inhibitor or angiotensin II receptor blocker (ARB), long-acting calcium channel blocker or thiazide diuretic. Obesity grade correlates with mortality risk secondary to cardiovascular disease; in fact, children with BMI >95th percentile have three- to five-fold increased risk of cardiovascular (CVD) mortality by age of 50 [56].

The first step in treating pediatric obesity should focus on lifestyle changes, particularly with a structured weight-management program and multidisciplinary approach [57]. Both intensive medical and lifestyle interventions for obesity treatment have demonstrated an average weight loss of around 5–15%, with variable results in compliance [58,59] and high percentage of drop out. Unfortunately, the probability that adolescents will attain a normal weight is very low and weight cycling was most common among subjects with baseline body mass indexes in the severe obesity category, as well as underdiagnosed binge eating that negatively impacts on treatment outcomes if not exhaustively addressed [60,61,62].

Weight loss interventions rarely resulted in substantial and sustained BMI reduction or resolution of co-morbidities for adolescents who have severe obesity [61,63].

Failure of lifestyle treatments and their risks for early complications of severe obesity poses bariatric surgery as the most effective weight loss treatment for severe obesity and its comorbidities in adolescents.

## 4. Eligibility Criteria for Bariatric Surgery

The use of bariatric surgery in children with severe obesity has received considerable recent attention, even if the surgery has not been widely accepted. In the adult population, combined multi-disciplinary interventions are often required in conjunction with bariatric surgery to achieve long-term satisfying results [64]. This is also reflected in the more recent trend to perform bariatric surgery as a strategy in adolescents with severe obesity [65]. However, many surgeons are still reluctant to embrace bariatric surgery in adolescent patients; they state that it is irreversible, invasive, and has potential life-long alterations [64]. Arguments in favor of an early surgical approach are based on the evidence that the efficacy of surgery is reduced over time and that adolescent obesity is more intractable and sustained than obesity in adults [66,67]. The recent American Society of Metabolic and Bariatric Surgery (ASMBS) guidelines outline the inclusion criteria for pediatric and adolescent bariatric surgery; with the significant improvement in long term data, they are less controversial than previous guidelines which often presented ethical concerns and scarcity of long-term data [58,68]. It must be noted that the criteria for being considered for metabolic and bariatric surgery as a pediatric patient are stricter than those used for adults. Bariatric surgery is proposed in adolescents with BMI ≥ 35 kg/m^2^ (moderate obesity) with major comorbidities or with a BMI ≥ 40 kg/m^2^ (severe obesity) with minor comorbidities [69].

The European Association for Endoscopic Surgery has recently developed a list of clinical practice guidelines on bariatric surgery which mirrors those proposed by ASMBS. Surgery should be considered for patients with BMI ≥ 40 kg/m^2^, for patients with BMI ≥ 35–40 kg/m^2^ with associated comorbidities and for patients with ≥ BMI 30–35 kg/m^2^ and type 2 diabetes and/or arterial hypertension with poor control despite optimal medical therapy [69].

According to the “Interdisciplinary European Guidelines on Metabolic and Bariatric Surgery”, BMI ≥ 40 kg/m^2^ (or 99.5th percentile for respective age) with associated comorbidities is not the only surgical criterion. 

BMI criterion must also be associated with all of the following:

- at least 6 months of lifestyle treatment for weight loss in a specialized center
- complete skeletal and sexual maturation
- ability to give informed consent with adequate understanding of the procedure
- ability to commit to comprehensive medical and psychological evaluation before and after surgery
- willing to participate in a post-surgery multidisciplinary program
- surgery access in a unit with specialist pediatric support

In 2015, the European Society for Paediatric Gastroenterology Hepatology and Nutrition (ESPGHAN) Position Statement provided additional criteria for surgery [70] in adolescents: BMI ≥ 40 kg/m^2^ with severe comorbidities (type 2 diabetes mellitus; moderate-to-severe sleep apnea, pseudotumor cerebri, NASH with advanced fibrosis—ISHAK score>1) or BMI ≥ 50 kg/m^2^ with mild comorbidities (e.g., hypertension, dyslipidemia, mild obstructive sleep apnea, chronic venous insufficiency, panniculitis, urinary incontinence, impairment in activities of daily living, NASH, gastroesophageal reflux disease, severe psychological distress, arthropathies related to weight). Moreover, patients are strongly recommended to avoid pregnancy for 1 year after surgery and are driven to adhere to nutritional guidelines after surgery.

According to the ASMBS guidelines, there are some contraindications for MBS:

- Obesity that can treated with medical therapy.
- Substance abuse within the past year.
- Current or planned pregnancy within 12–18 months of the scheduled surgical procedure.
- Concomitant eating disorders.
- Medical, psychiatric, psychosocial issues interfering with postsurgical recommendations and required lifestyle modifications

It is worth noticing that adolescents represent a vulnerable group of patients in a very susceptible transitional developmental stage of self-concept, influenced by relationships, social environment [71], and the educational system. As described above, adolescents with obesity often suffer from body dissatisfaction, low self-esteem, teasing, and symptoms of mood deflection in combination with a history of eating disorder symptoms such as binge eating (BE) that may support, or even worsen, the vicious cycle of obesity which could lead to negative outcomes in metabolic and bariatric surgery [72]. Loss of control (LOC) with snacking and binge eating (BE) is prevalent among adolescents (15–28%) seeking bariatric surgery [73], even if few studies have investigated prevalence of LOC and BE in this category of subjects [74,75]. Multidisciplinary assessment involving psychological evaluation is mandatory before surgery [76] and ASMBS pediatric guidelines recommend preoperative assessment for LOC and treatment with systemic family-based therapy, individual cognitive behavioral therapy, and, if necessary, medication [77].

## 5. Laparoscopic Sleeve Gastrectomy as Preferred Surgical Approach 

Bariatric surgery options include Roux-en-Y gastric bypass (RYGB), biliopancreatic diversion with duodenal switch (BPD-DS), sleeve gastrectomy (SG) and adjustable gastric banding (AGB). 

According to the pediatric metabolic and bariatric surgery guidelines, vertical sleeve gastrectomy (VSG) has become the most commonly used and recommended surgery procedure in adolescents with severe obesity, because of its near-equivalent weight loss and efficiency regarding co-morbidities to the RYGB, with fewer revision surgeries and better nutrient absorption [58].

A minimally invasive surgical approach has become the preferred technique in bariatric surgery. Laparoscopic sleeve gastrectomy (LSG) initially represented the first stage of the duodenal switch procedure in patients with severe obesity, but it has been rapidly used as a single procedure due to its technical ease and good results [78,79]. 

SG has recently gained attention both in the adult and adolescent bariatric population due to several longitudinal studies which demonstrate excess weight loss between 38% and 83%, and it is less technically complex when compared to RYGB with less malabsorption of specific nutrients [80,81,82]. Initial results of SG in adolescents are encouraging; they demonstrate results similar to adult studies and SG is the predominant choice in centers offering bariatric surgery within this age group [83,84,85,86].

LSG is considered the most commonly performed bariatric surgery worldwide. The safety of the LSG technique, together with high survival rates have been widely demonstrated and LSG has become the surgery procedure of first choice in patients with severe obesity worldwide both in adulthood and pediatric age [79,87,88,89,90,91,92], and in the adolescent population, LSG appears to have a higher safety profile than other bariatric operations [93]. The effectiveness of a single procedure in weight loss has been demonstrated in pediatric age [88].

LSG consists of forming a “sleeve” from the stomach by surgically stapling its edges. This technique is also referred to as “greater curvature gastrectomy”, or “vertical gastrectomy” or “pylorus preserving gastric tube”. The vertical resection involves the greater curvature and the fundus and leads to a gastric tuberization [88] Gastric resection includes approximately 80% of the stomach and the remnant gastric area has a capacity > 100 mL. Gastric reduction does not require a gastrointestinal anastomosis or bypass which makes it easier than RYGB or BPDDS [94,95].

This minimally invasive approach has become the preferred technique in bariatric surgery and the laparoscopic approach is commonly adopted in children and adolescents as the treatment of choice. Robotic surgery presents many advantages over the laparoscopic approach such as improvement of surgical ergonomics, shortened length of stay, decreased tissue damage, and decreased postoperative need of analgesia [96]. 

With regards to minimally invasive bariatric surgery, specific laparoscopic instrumentation must be used which includes longer trocars and longer instruments, and bougie devices (34–36 F) useful to accommodate the thicker abdominal wall of patients with obesity. The patient is placed in the supine position, 20′ tilted in reverse Trendelenburg. The procedure is performed with four trocars: a 12 mm trocar in the umbilicus for the 30-degree laparoscope, two operative trocars (5 and 15 mm) to the left and right of the umbilicus and a 5 mm trocar in the left hypo-condrium (or subxiphoid area) for liver retraction. The surgeon stands between the legs of the patient. 

As first step, the dissection starts from the cardial region, dividing the peritoneal sheet and removing the fat in front of the hiatus until the left crus is exposed from behind. The dissection should proceed distally to the antrum. The site of the distal transection should preserve the antrum and should be placed nearly 2–6 cm proximal to the pylorus. The gastrocolic ligament is dissected with a vessel sealing device (Maryland Ligasure^®^) starting halfway at the greater curve. It is important to stay close to the gastric wall to avoid damaging surrounding tissues and vessels and to reduce the risk of portal vein thrombosis. Once the lesser sac is partially opened the posterior wall of the stomach is inspected and the dissection is extended posteriorly to take down all adhesions.

The dissection proceeds inferiorly to reach the marked point on the antrum, and superiorly, up to the cardia. The stapled sleeve gastrectomy is performed with an EndoGIA starting from the distal part. The bougie (34–36 F) is used after the first fire as a guide inside the stomach lumen (inserted from the mouth and directed to a point distal to the divided omental attachments, under laparoscopic surveillance). Gastrectomy is completed with two–four vertical firings. The last firing is performed with a lateral movement to partially maintain the gastro-phrenic ligament, to preserve the vascularization and to keep part of the muscular fibers over the cardia for gastro-esophageal junction competence (1 cm left of the gastro-esophageal junction, leaving a sort of “dog ear”). At the end, it is possible to complete the vessel dissection by dividing the short vessels. The air insufflation test can be performed to rule out a torsion of the sleeve. The specimen is then extracted from the umbilical access.

Data on short and middle term outcomes in pediatrics show no major complications and a low rate of minor complications (4.3% according to Alqahtani et al.) [13] after LSG, with no evidence of mortality [97,98,99].

Retrospective analysis from the Metabolic and Bariatric Surgery Accreditation and Quality Improvement Program (MBASQIP) database (USA) confirmed that both LSG and RYGB are relatively safe, but the LSG is associated with a significantly lower rate of major complications in the first month after surgery [100] and with shorter operative times [95,101], especially in accredited centers [93]. 

In 2016, Pepper et al. [96] reported the results of a retrospective analysis showing the same results when comparing LSG and robotic sleeve gastrectomy in terms of safety and efficacy. Shorter length of stay and better postoperative pain control were considered potential benefits of the robotic approach [102]. 

As reported in Table 3, surgical complications are not excluded. In the immediate postoperative period, nausea, vomiting and dehydration, anastomotic leak and gastric tube twist as well as volvulus may occur; wound infection at the trocar site is also a recurrent complication described in patients with severe obesity (0.6%). In middle term outcome, major complications are described, but not recorded in pediatrics [13]. Long-term follow up is recommended for gastroesophageal reflux disease secondary to the risk of developing esophageal disease such as Barrett’s esophagus. 

## 6. Nutritional Status and Nutritional Risks

The primary objective of the pre-surgery investigation is to assess the candidacy of the subject through a complete and multidisciplinary medical, surgical and psychological evaluation, not only of whether the patient meets inclusion criteria but also to establish which procedure is most optimal [103]. Pre-surgery assessment also represents an opportunity to provide counselling and anticipatory guidance on topics including eating habits and behavior. To reach successful results after surgery, patients must optimize their lifestyle habits, make healthy dietary choices, meet nutritional requirements, adhere to a robust physical activity level, and learn to sense their “new” needs in feeling hunger and satiety [104].

For this reason, preparation for bariatric surgery should include counselling of both patients and their caregivers, to modify any deleterious behaviors which may coexist with obesity such as smoking, sleep disturbances, and sedentary behavior. In a recent study [18,105] conducted on adolescents with severe obesity enrolled in the Teen-LABS study, physical activity appeared to further reduce cardiovascular risk and support long-term weight loss maintenance after bariatric surgery. On the other hand, the easy availability and access of energy dense fast foods, such as sugary beverages and snacks, contributes to weight gain and this in turn promotes sedentary lifestyle, fueling a vicious cycle [106] with weight regain even after bariatric procedure [107].

Adolescents seeking bariatric surgery and their families must also be counseled on the key role of supplements that need to be regularly taken in order to reduce the risk of nutrient deficiencies after the metabolic and bariatric surgical procedure [77], along with a healthy and personalized diet, which considers both growth requirements and surgery limitations with regards to portion sizes and food tolerance concerns.

In order to properly tackle the consequent risk of malnutrition, a pre-surgery nutritional status assessment is highly recommended, including biomarkers such as serum iron, folate, ferritin, and total iron-binding capacity (TIBC); thiamin (B1); vitamin B12 and B6; calcium, Parathyroid Hormone (PTH), alkaline phosphatase, vitamin D, phosphorus, calcium, magnesium, potassium and zinc [77]. It is worth considering that while the 2018 ASMBS pediatric guidelines suggest universal preoperative testing of B1, B12, and folate, the reported prevalence of deficiency of these micronutrients in adolescents with severe obesity is 0–1%, 0–1%, and 0–4%, respectively [99,100].

Conversely, vitamin D, which is known to play a role in bone health and metabolism throughout lifespan, is frequently deficient in individuals with obesity; a European study found around 39% prevalence of 25(OH)D deficiency (< 10 ng/mL) among adolescents [108], while in a recent prospective [109,110] cohort study on pre- and post-bariatric surgery adolescents, basal levels of 25-hydroxyvitamin D were insufficient in 37% of participants (*n* = 242). The pandemic increase in obesity is inversely associated with vitamin D levels, which is known to be stored in fat tissue, resulting in an increased risk of impaired glycemic control and metabolic syndrome in the general population [111].

One of the primary considerations in bariatric surgery for pediatric patients is the impact on physical growth/maturation, and the compliance with postsurgical medical nutrition therapy which requires continuous care to monitor patient’s progress, as well as continuous reinforcement of motivation. After bariatric procedures, dietary patterns need particular attention since adolescents are at higher risk of nutritional complications than adults [80]. The post-surgery diet should focus on adequate protein intake, hydration, and vitamin-mineral supplementation.

Sleeve gastrectomy is often the first choice surgery in adolescents, mainly because it is more conservative and less related to nutritional status impairment, although partial gastrectomy leads to loss of the gastric body and fundal parietal cell, as well as a marked decrease in the production of gastric acid, which affects iron, Vitamin B12, and folate absorption [112], Table 1.

Results confirmed by a cohort study on 242 adolescents, including 67 with sleeve gastrectomy and 161 with gastric bypass, showed a significant decrease in vitamin B12 levels in both groups after 3 years surgery [113]. Evidence revealed that sleeve gastrectomy impacts less on postoperative vitamin B12 deficiency risk than gastric bypass, while neither surgical procedure showed any particular differences with regard to the risk of postoperative anemia and iron deficiency [113]. A recent systematic review [114] analyzed the outcomes of bariatric surgery in adolescents with a follow-up ≥ 5 years, which revealed a high prevalence of iron deficiency and anemia; however, only gastric bypass procedure was considered. The previously mentioned study conducted by Inge et al. [83] found hypo-ferritinemia in 57% of individuals 3 years after the bariatric procedure with over 10% in adolescents who underwent sleeve gastrectomy.

A recent retrospective review by Goldebrg et al. [115] aimed to investigate if standard multivitamin supplementation is sufficient to prevent anemia in adolescents undergoing sleeve gastrectomy with biomarkers including iron, ferritin, folate, vitamin B12, hemoglobin and hematocrit, 3, 6 and 12 months after surgical intervention. Patients’ folate levels were lower 3- and 6-months post-surgery compared to baseline, but returned to baseline levels at 12 months. Vitamin B12 levels were lower 6 months post-surgery compared to 3 months, though not different from levels at T0, and returned to baseline levels at 12 months. There were no changes in iron, ferritin, Hgb or Hct during the 1-year follow up.

Other authors [116] found low ferritin level in 33.3% of adolescents (*n* = 79), 2 years after sleeve gastrectomy, while low hemoglobin levels were found only in females. They also found a high prevalence of vitamin D and albumin deficiency (89.3% and 38% respectively) as well as hypo-ferritinemia. Standardized nutritional supplements were provided to all patients and these included daily multivitamin tablets with vitamins A (retinol) C, D3, E K, B1 (thiamine), B2, B3, B5, B6 (pyridoxine), B8, B12 (cobalamin), folate and minerals including chrome, iron, magnesium, selenium, and zinc. In this study subjects with vitamin and/or mineral deficiencies in the preoperative assessment received treatment accordingly prior to surgery. The percentage of adolescents with hypoalbuminemia increased to 38.3% post-surgery and this is another important nutritional outcome that needs to be considered [116].

Hypoalbuminemia may occur especially during the first few months post-surgery [117]; it is important to consider that muscle mass loss is a negative phenomenon, as non-adipose tissue is responsible for most of the resting metabolic rate, regulation of body temperature and weight maintenance [118]. There are still no data available on vegetarian/vegan dietary patterns after bariatric surgery in adolescence, but it is important to guarantee an optimal protein intake with a balanced diet, and supplementation must be prescribed while educating individuals on ideal eating after metabolic and bariatric surgery and to minimize nausea and vomiting. Persistent vomiting is a common symptom after bariatric surgery, and this symptom is associated with nausea and a loss of appetite which is also a non-specific symptom of thiamine deficiency [119]. Thiamine (vitamin B1) is essential in multiple metabolic pathways, including the extraction of ATP from glucose and the generation of precursors in brain metabolism [120] and its deficiency must be accurately diagnosed.

Tang et al. [121] recently performed a retrospective study on 147 bariatric patients between 18 and 65 years old who underwent SG to evaluate thiamine deficiency. Basal thiamine level before surgery was below the cut off in 25.7% of subjects enrolled while thiamine deficiency was present in approximately 20% of the subjects enrolled at all follow-ups (3 months, 6 months, 1 year).

Subjects with thiamine deficiency were also more likely to report nausea (*p* = 0.002), vomiting (*p* = 0.001), and decreased appetite (*p* = 0.017).

In addition to nutrients, it is worth considering another often-undervalued issue: water consumption. Dehydration in one of the most common reasons for readmission of minor complications in adolescents after metabolic and bariatric surgery [122]. They should be advised to consume liquids slowly, ideally 30 min after meals, to prevent gastrointestinal issues, and in adequate amounts to maintain hydration.

## 7. Weight Loss and Resolution of the Metabolic Comorbidities

Bariatric surgery induces significant weight loss for adolescents with severe obesity. Weight loss is much greater in patients after bariatric surgery compared to patients treated with lifestyle interventions [123,124,125]. According to Roberts [126], The mean decrease in BMI across studies in which this endpoint was assessed was 29% (CI 24–37.5%); mean weight loss, when measured, was 27% (25–29%). In a meta-analysis reported by Black [123], 637 patients from 23 studies showed a significant decrease in BMI at 1 year (average weighted mean BMI difference: −13.5 kg m −2; 95% confidence interval− 14.1 to −11.9); BMI loss after SG resulted in a mean BMI reduction of −14.5 kg m −2; 95%CI −17.3, 11.7) [123].

**Figure 1:**
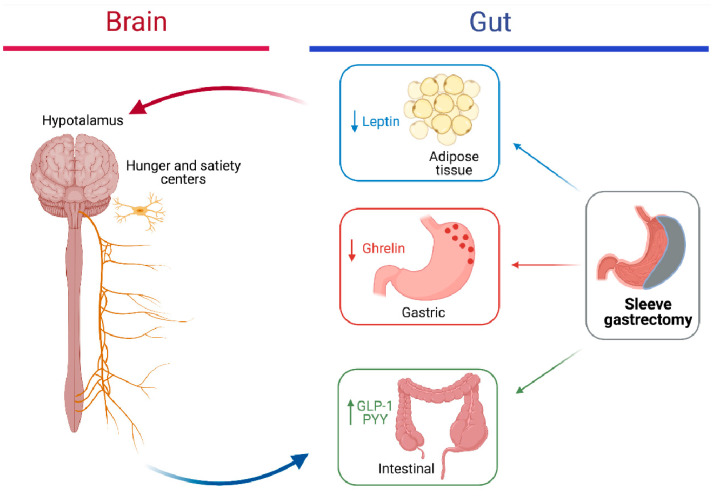
Potential mechanisms of sleeve gastrectomy for reducing body weight and improving metabolism. GLP-1: glucagon-like peptide 1; PYY: peptide YY.

The mechanisms implicated in the long-term weight loss and resolution of the complications after bariatric surgery include mechanical restriction (related to the reduction in stomach size) and malabsorption, as well as changes in gut hormones, brain regulation of appetite and satiety, involving endogenous molecules such as ghrelin, glucagon-like peptide 1 (GLP-1) and peptide YY, leptin [78,127,128], Figure 1.

Weight loss is favored by the removal of most of the stomach’s greater curvature resulting in a tubular, smaller stomach made by the lesser curvature, resistant to stretching. The preservation of the pylorus helps in regulating gastric emptying (GE) which contributes to satiety [128,129].

Neuroendocrine changes are likely to be involved in weight loss as well as in metabolic comorbidity resolution. All the metabolic improvements achieved post bariatric surgery have led to the creation of the term metabolic surgery or metabolic-bariatric surgery (MBS). The digestive-absorptive process starts with the so called “cephalic phase”, which results from the intersection of senses such as sight, smell or taste, followed by the release of many hormones, including ghrelin, insulin, pancreatic polypeptide (PP), and gastrin via vagal mechanism [130]. Ghrelin is a small peptide hormone released by the stomach, which stimulates food intake, acting as an endogenous ligand for its receptor, the growth hormone secretagogue receptor. SG and the other procedures involving gastrectomy or partial gastrectomy lower ghrelin levels, removing ghrelin-producing cells. Nevertheless, we have to remember that levels tend to recover after partial gastrectomy [131,132].

PP is an anorexigenic hormone secreted by specific cells in the pancreatic islets, in response to food stimulation involving the vagal system; it is usually reported to be unchanged after MBS [133,134]. Gastrin, on the other hand, which is secreted by the G-cells of the antrum, aids in gastric acid secretion and has been suggested to act in facilitating insulin secretion via gastrin receptors in the pancreatic islets. Nonetheless, there are few data on serum gastrin levels after bariatric surgery, which have provided mixed results [133,134,135,136,137].

GLP-1 (glucagon-like peptide 1), produced by the L-cells in the distal segments of the small intestine and colon, stimulates insulin secretion and inhibits glucagon along with gastrointestinal secretions and motility. Its levels during oral glucose or meal stimulation have been shown to be permanently increased after SG [135].

There are animal and human studies showing that postprandial levels of GLP-1 are enhanced after SG, which suggests the importance of such alterations in intestinal hormone secretion [138,139,140,141].

Peptide YY (PYY) also known as peptide tyrosine, secreted (as well as GLP-1) by mucosal L-cells in the small and large intestine, inhibits gastric, pancreatic and intestinal secretions. Its effects on gastrointestinal motility are still unanswered, even if there are studies reporting increased postprandial levels after MBS, which could ultimately lead to enhanced satiety [138,139,140,141,142,143,144,145].

Cholecystokinin or *CCK*, is a neuropeptide hormone produced by intestinal I-cells that regulates pancreatic enzyme secretion, inhibits gastric emptying and gastrointestinal motility, also acting as a satiety signal. CCK levels have been reported to increase after MBS [146,147,148].

Changes in neuroendocrine profile are associated with long-term weight reduction and resolution of comorbidities after gastric surgery [133,149,150]. Some forms of diabetes such as type 2 diabetes, have been shown to improve after MBS (including SG), which does not bypass the duodenum, yielding GLP-1increases, weight loss and improvements in glucose metabolism [138].

Data from the national database for the American Society for Metabolic and Bariatric Surgery (ASMBS) Center of Excellence program reported a high 12-months diabetes remission rates in adults [151].

The STAMPEDE [152] trial conducted among adult patients with obesity with uncontrolled type 2 diabetes (randomization of 150 patients with uncontrolled T2DM to intensive medical therapy alone or intensive medical therapy plus RYGB or SG) showed that intensive medical therapy + bariatric surgery achieves adequate glycemic control of type 2 DM in patients with obesity, better than medical therapy alone. At 36 months, the primary endpoint (HbA1c level 6%) was achieved by 5% of patients in the medical group compared with 24% in the SG arm. In addition, the use of anti-diabetic therapy was lower when compared to the medical therapy cohort. Patients who underwent surgical treatment showed improved glycemic control at 3 years. Weight loss and shorter duration of diabetes were the main predictors of HbA1c level of 6% after surgery [152].

A recent study that evaluated weight loss up to four years after VSG showed greater benefits in adolescents than in adults with T2D resolution after MBS (both VSG and RYGB) in 87% of adolescents versus 54% of adults [67], therefore suggesting strong advantages of early surgery.

Regarding inflammatory markers, studies demonstrated that there is a remarkable reduction in CRP and urinary cytokines levels, signifying that MBS also improves systemic and renal inflammatory status [153,154].

In regard to the cardiovascular system, obesity is a well-known chronic degenerative disease that remarkably decreases life expectancy. Sleeve surgery for weight loss has proven to increase life expectancy which reduces mortality for cardiovascular diseases in patients with severe obesity showing that the total amount of cardiovascular diseases, myocardial infarction, stroke, and systolic blood pressure (SBP) significantly reduced about ten years after SG procedures [155,156,157]. Various studies have shown that high blood pressure is resolved after SG [155,156,157,158].

Another study demonstrated 74% remission rate for hypertension and 66% for dyslipidemia in adolescent subjects [159]. 

The literature also reports antihypertensive drug reduction in almost 59% of patients who underwent VSG. Another study demonstrated resolution of hypertension after VSG in adolescents compared to adults, showing the effectiveness of MBS if performed at a young age [67,160].

The modifications of gastric emptying may also explain some metabolic changes. Braghetto et al. found that at 3 months after SG, GE became significantly accelerated for both solid and liquid meals versus controls [161]. Another group reported faster GE after solid meals at 6- and 24-months post SG in adult subjects. Faster gastric emptying and secretion to the distal intestinal segments could enhance gut hormone secretion contributing to increased weight loss and improved glucose homeostasis. 

## 8. Future Perspectives

Childhood obesity must be treated with urgency given the significant individual and societal costs. Because severe obesity can begin early, prevention should focus on promoting a healthy lifestyle in the prenatal, neonatal, and early childhood years when nutritional choices can affect long-term chronic disease risk [162]. Further research into the biology, neuro-endocrinology and psychology of weight-loss maintenance should be undertaken to develop more effective medical and surgical approaches. Future perspectives in the management of obesity in children should include patient-tailored surgery, limited to the gastric surface involved in the neuroendocrine gut system and appetite control. A multidisciplinary approach is necessary to study long term postoperative benefits and complications in adulthood, including musculoskeletal development, fertility preservation, psycho-social attitude and cognitive and behavioral skills.

## 9. Conclusions

Bariatric surgery should be considered in adolescents with moderate to severe obesity who have previously been treated with a lifestyle medicine approach with unsuccessful results. Bariatric surgery, and in particular sleeve gastrectomy, is an effective method for weight loss and its maintenance over time, providing comorbidities resolution and a better quality of life in adulthood [163] However, there are many perceived patient, provider, and health system barriers to bariatric surgery. Furthermore, more must be done in order to ensure long-term treatment efficacy, allow healthy growth and prevent nutritional status impairment as well as inadequate weight loss and weight regain.

Multidisciplinary pre- and post-operative care is recommended, including adequate medical nutrition, behavioral and pharmacological treatment, for the continuous care of the adolescent patient candidate for metabolic and bariatric surgery. Only in this way will it be possible to ensure adequate growth and a healthy adult life via permanent lifestyle changes.

## Figures and Tables

**Table 1:** Obesity related co-morbidities in children and adolescents. Kansra et al. [18], modified.

**Cardiovascular** Hypertension Dyslipidemia	**Endocrinology** Type II Diabetes Mellitus Precocious puberty Insulin resistance PCOS Menstrual irregularities
**Gastrointestinal** Gastroesophageal reflux disease Gallstones Non-alcoholic fatty liver disease	**Orthopedics** Slipped capital femoral epiphysis Ankle sprains Blount’s disease Arthritis Join pain Tibia vara Flat feet
**Neurological** Pseudotumor cerebri Headache	**Renal** Glomerulonephritis Nephrotic Syndrome
**Respiratory** Asthma Obstructive sleep apnea	**Dermatological** Acanthosis Nigricans Striae Hidradenitis Suppurativa
**Psychological** Depression Anxiety Poor-self-Esteem Poor Body Image Eating disorder Sleep Disturbance	

**Table 2:** Diagnostic criteria for metabolic syndrome (MetS) in adolescent children aged 10 to 16 years according to International Diabetes Federation (IDF) versus IDEFICS study criteria, those recommended by Cook et al. [20], and those proposed by De Ferranti et al. [21].

International Diabetes Federation	IDEFICS Study	Cook et al.	de Ferranti et al.
Waist circumference ≥90th percentile for age and sex associated with at least 2 of the following: (1) Fasting blood glucose ≥100 mg/dL (≥5.6 mmol/L) (2) Triglyceride level ≥150 mg/dL (≥1.7 mmol/L) (3) HDL cholesterol ≤40 mg/dL (4) Systolic blood pressure ≥130 mmHg or diastolic blood pressure ≥85 mmHg	≥3 of the 4 following criteria: (1) waist circumference ≥90th percentile (monitoring level) or ≥95th percentile (action level) (2) Systolic and/or diastolic blood pressure ≥90th percentile (monitoring level) or ≥95th percentile (action level) (3) Triglycerides ≥90th percentile (monitoring level) or ≥95th percentile (action level) or HDL cholesterol ≤10th percentile (4) HOMA-IR or fasting plasma glucose ≥90th percentile (monitoring level) or ≥95th percentile (action level)	≥3 of the 5 criteria below: (1) waist circumference ≥90th percentile (2) Blood Pressure ≥90th percentile (3) Triglycerides ≥110 mg/dL (4) HDL-cholesterol ≤40 mg/dL (5) Impaired fasting glucose (≥110 mg/dL)	≥3 of the 5 criteria below: (1) waist circumference ≥75h percentile (2) Blood Pressure ≥90th percentile (3) Triglycerides ≥100 mg/dL (4) HDL-cholesterol ≤50 mg/dL (5) Impaired fasting glucose (≥110 mg/dL)

IDEFICS: Identification and prevention of dietary- and lifestyle-induced health effects in children and infants. HDL: High-density lipoprotein. HOMA-IR: Homeostatic model assessment fo insulin resistance.

**Table 3:** Surgical and medical complications after laparoscopic sleeve gastrectomy (LSG).

General Complications	Insufficient Weight Loss and Weight Regain
Surgical complications (mostly minor complications)	Acute post-operative − Nausea, vomiting and dehydration − Anastomotic leak (unexplained tachycardia within the first post-operative week) − Gastric tube twist and volvolus − Wound infection at trocar site − At middle/long-term follow-up − Hiatal hernia − Gastroesophageal reflux − Barrett’s esophagus − Stricture (rare)
Nutritional risks	Micronutrient deficiencies − Iron deficiency and anemia − Vitamin B12 deficiency − Reduction of folate absorption − Hypo-ferritinemia − Hypoalbuminemia − Thiamine (vit B1) deficiency Dehydration Adverse bone density and bone microarchitectural changes

## Data Availability

Not applicable.
